# Encephalomyocarditis Virus Viroporin 2B Activates NLRP3 Inflammasome

**DOI:** 10.1371/journal.ppat.1002857

**Published:** 2012-08-09

**Authors:** Minako Ito, Yusuke Yanagi, Takeshi Ichinohe

**Affiliations:** Department of Virology, Faculty of Medicine, Kyushu University, Maidashi, Higashi-ku, Fukuoka, Japan; University of North Carolina at Chapel Hill, United States of America

## Abstract

Nod-like receptors (NLRs) comprise a large family of intracellular pattern- recognition receptors. Members of the NLR family assemble into large multiprotein complexes, termed the inflammasomes. The NLR family, pyrin domain-containing 3 (NLRP3) is triggered by a diverse set of molecules and signals, and forms the NLRP3 inflammasome. Recent studies have indicated that both DNA and RNA viruses stimulate the NLRP3 inflammasome, leading to the secretion of interleukin 1 beta (IL-1β) and IL-18 following the activation of caspase-1. We previously demonstrated that the proton-selective ion channel M2 protein of influenza virus activates the NLRP3 inflammasome. However, the precise mechanism by which NLRP3 recognizes viral infections remains to be defined. Here, we demonstrate that encephalomyocarditis virus (EMCV), a positive strand RNA virus of the family *Picornaviridae*, activates the NLRP3 inflammasome in mouse dendritic cells and macrophages. Although transfection with RNA from EMCV virions or EMCV-infected cells induced robust expression of type I interferons in macrophages, it failed to stimulate secretion of IL-1β. Instead, the EMCV viroporin 2B was sufficient to cause inflammasome activation in lipopolysaccharide-primed macrophages. While cells untransfected or transfected with the gene encoding the EMCV non-structural protein 2A or 2C expressed NLRP3 uniformly throughout the cytoplasm, NLRP3 was redistributed to the perinuclear space in cells transfected with the gene encoding the EMCV 2B or influenza virus M2 protein. 2B proteins of other picornaviruses, poliovirus and enterovirus 71, also caused the NLRP3 redistribution. Elevation of the intracellular Ca^2+^ level, but not mitochondrial reactive oxygen species and lysosomal cathepsin B, was important in EMCV-induced NLRP3 inflammasome activation. Chelation of extracellular Ca^2+^ did not reduce virus-induced IL-1β secretion. These results indicate that EMCV activates the NLRP3 inflammasome by stimulating Ca^2+^ flux from intracellular storages to the cytosol, and highlight the importance of viroporins, transmembrane pore-forming viral proteins, in virus-induced NLRP3 inflammasome activation.

## Author Summary

The innate immune system, the first line of defense against invading pathogens, plays a key role not only in limiting microbe replications at early stages of infection, but also in initiating and orchestrating antigen-specific adaptive immune responses. The innate immune responses against viruses usually rely on recognition of viral nucleic acids by host pattern-recognition receptors such as Toll-like receptors and cytosolic helicases. In addition, recent studies have indicated that certain viruses activate the NLRP3 inflammasome, a multiprotein complex containing the intracellular pattern-recognition receptor NLRP3, which in turn induces secretion of proinflammatory cytokines. We have previously revealed the role of the NLRP3 inflammasome in innate recognition of influenza virus, in which the influenza virus proton-selective ion channel M2 protein, but not viral RNA, is required. Here, we demonstrate that another RNA virus, encephalomyocarditis virus (EMCV), also activates the NLRP3 inflammasome in a viral RNAindependent manner. Instead, the EMCV viroporin 2B, which is involved in Ca^2+^ flux from intracellular storages into the cytosol, activates the NLRP3 inflammasome. Our results highlight the importance of viroporins, virusencoded transmembrane pore-forming proteins, in recognition of virus infections by NLRP3.

## Introduction

The innate immune system, the first line of defense against pathogens, utilizes pattern-recognition receptors (PRRs) to detect pathogen-associated molecular patterns (PAMPs). RNA viruses are detected by host PRRs including Toll-like receptors (TLRs), retinoic acid-inducible gene-I (RIG-I)-like helicases (RLHs), and Nod-like receptor (NLR) family, pyrin domain-containing 3 (NLRP3) [1], [2], [3], [4].

NLRP3 plays an important role in the secretion of proinflammatory cytokines interleukin 1 beta (IL-1β) and IL-18 after viral infections. Upon activation, NLRP3 forms the protein complex termed “NLRP3 inflammasome” by recruiting the apoptosis-associated speck-like protein containing a caspase recruitment domain (ASC) and pro-caspase-1, which is activated by autocatalytic cleavage within the complex [5]. The active caspase-1 catalyzes proteolytic processing of pro-IL-1β and pro-IL-18 into active cytokines that are then released across the plasma membrane by poorly understood mechanisms [6]. Secretion of these two cytokines requires upregulations of pro-IL-1β, pro-IL-18, and NLRP3, which are induced by signals from TLRs, IL-1 receptor or tumor necrosis factor receptor (signal 1), in addition to the activation of caspase-1 through inflammasome activation (signal 2) [7], [8]. In influenza virus infection, the signal 1 is provided by TLR7 that recognizes influenza virus RNA, whereas the signal 2 comes from the function of the virus-encoded proton-selective ion channel M2 protein, but not from viral RNA [9].

Encephalomyocarditis virus (EMCV), a member of the genus *Cardiovirus* within the family *Picornaviridae*, is a nonenveloped, positive single-stranded RNA virus. This virus has a ∼7.8 kb viral genome covalently linked to a viral protein VPg at its 5′ end that serves as a primer for viral RNA synthesis. The viral genome encodes a polyprotein precursor, which is divided into the P1, P2 and P3 regions and processed mainly by the virus-encoded 3C protease. Processing of the P1 region produces the structural capsid proteins 1A (VP4), 1B (VP2), 1C (VP3), and 1D (VP1), whereas the P2 and P3 regions are processed into the nonstructural proteins 2A, 2B, 2C, 3A, 3B (VPg), 3C and 3D as well as cleavage intermediates (2BC, 3AB, and 3CD) [10]. The EMCV 2A protein has been implicated in the shutoff of host protein synthesis and viral pathogenesis [11], [12], but little is known about the roles of the EMCV 2B and 2C proteins. Picornavirus 2B proteins have been reported to act as viroporins, transmembrane pore-forming viral proteins that alter the membrane permeability to ions by forming membrane channels, and participate in a range of viral functions [13]. The avian encephalomyelitis virus (AEV) 2C protein is known to induce apoptosis [10], [14].

EMCV is detected by at least three classes of PRRs in the host innate immune system. First, double-stranded RNA (dsRNA) produced in EMCV-infected cells is recognized by TLR3 [15]. Second, EMCV RNA is recognized by melanoma differentiation associated gene 5 (MDA5), a member of RLHs, unlike many other RNA viruses which are recognized by RIG-I [16], [17], [18]. Activation of RLHs leads to the induction of type I interferons in infected cells. Third, the NLRP3 inflammasome detects EMCV by unknown mechanisms [19], [20]. In this study, we examined how EMCV activates the NLRP3 inflammasome. Our results indicate that EMCV increases the local Ca^2+^ concentration in the cytoplasm by stimulating Ca^2+^ flux from intracellular storages through the action of its viroporin 2B, thereby activating the NLRP3 inflammasome. Together with the data with the influenza virus M2 protein, our study reveals the importance of viroporins in virus-induced NLRP3 inflammasome activation.

## Results

### EMCV activates the NLRP3 inflammasome

**Figure 1:**
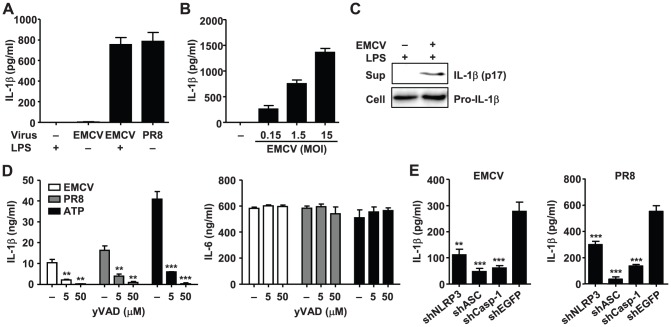
NLRP3 inflammasome activation by EMCV. (A) BMMs were infected with EMCV or influenza virus PR8 in the presence or absence of LPS at an MOI of 1.5. (B) LPS-primed BMMs were infected with EMCV at the indicated MOIs. (C) Immunoblot analysis of the mature (p17) form of IL-1β in the supernatants (Sup) and pro-IL-1β in extracts (Cell) of LPS-primed BMDCs infected with EMCV. (D) LPS-primed BMDCs were treated with EMCV, influenza virus PR8 or ATP in the presence or absence of yVAD-CHO. (E) LPS-primed RAW264.7 cells stably expressing shRNA against NLRP3, ASC, caspase-1, or EGFP mRNAs were infected with EMCV or influenza virus PR8. Cell-free supernatants were collected at 24 h after infection and analyzed for IL-1β or IL-6 by ELISA. Data are representative of at least three independent experiments, and indicate the mean ± S.D. (A, B, D, and E). **, *P*<0.01; ***, *P*<0.001.

In order to determine whether EMCV infection activates the inflammasomes, we measured IL-1β secretion from unprimed or lipopolysaccharide (LPS)-primed mouse bone marrow-derived macrophages (BMMs) infected with EMCV or influenza virus PR8 (Figure 1A). LPS induces pro-IL-1β in the cytosol (signal 1) [7]. In agreement with a previous report [20], EMCV infection induced release of IL-1β from LPS-primed BMMs, but not from unprimed BMMs. The amount of IL-1β secretion from BMMs was increased in a multiplicity of infection (MOI)-dependent manner (Figure 1B). Western blot analysis demonstrated that the p17 subunit, the mature processed form of IL-1β, was secreted in the supernatant (Figure 1C). Furthermore, the secretion of IL-1β after EMCV infection was inhibited by yVAD-CHO, a specific peptide inhibitor of caspase-1, without affecting the secretion of IL-6 in both bone marrow-derived dendritic cells (BMDCs) and BMMs (Figure 1D and Figure S1). To dissect the importance of various components of the inflammasome complex in IL-1β secretion in response to EMCV infection, we generated RAW264.7 cells stably expressing short hairpin RNA (shRNA) against murine NLRP3, ASC or caspase-1. Quantitative RT-PCR and Western blot analysis confirmed knockdown of NLRP3, ASC, and caspase-1 at the mRNA and protein levels (Figure S2A–B). Furthermore, these cells produced comparable levels of IL-6 after LPS stimulation to EGFP-knockdown cells (Figure S2C), indicating that innate immunological responses are not generally affected by the knockdown. Like that after influenza virus infection [9], IL-1β secretion after EMCV infection was found to be dependent on NLRP3, ASC and caspase-1 (Figure 1E), indicating that EMCV infection activates the NLRP3 inflammasome. This is consistent with previous studies demonstrating that EMCV-induced IL-1β production was abrogated in NLRP3^−/−^, ASC^−/−^, and caspase-1^−/−^ BMDCs [19], [20].

### Viral RNA is insufficient to trigger NLRP3-mediated IL-1β secretion

**Figure 2:**
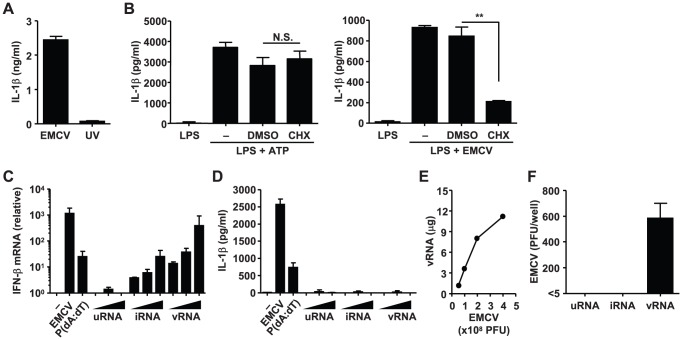
Viral RNA-independent recognition of EMCV by the NLRP3 inflammasome. (A) LPS-primed BMDCs were inoculated with live or UV-inactivated EMCV. (B) BMDCs were primed with LPS for 3 h, and then treated with 100 µM of cycloheximide (CHX) for 1 h prior to the treatment with ATP or infection with EMCV. (C, D) BMMs were infected with EMCV or transfected with 10 µg/ml poly(dA∶dT) or various amounts (12.5, 25, 50 µg) of total RNA from uninfected cells (uRNA), total RNA from EMCV-infected cells (iRNA), or EMCV genomic RNA (vRNA) for 18 h. Total RNA was extracted from virus-infected or RNA-transfected BMMs. IFN-β mRNA levels were assessed by RT quantitative PCR. GAPDH was used as an internal control. Data are pooled from three independent experiments (C). Supernatants were analyzed for the presence of mature IL-1β by ELISA (D). (E) Viral genomic RNAs were extracted from indicated amounts of EMCV, and their concentrations were determined. (F) Virus titers in the supernatants of the cells examined in (D) are shown. Data are representative of at least three independent experiments, and indicate the mean ± S.D. N.S., not significant. **, *P*<0.01.

We next examined whether viral replication is required for NLRP3 inflammasome activation by EMCV. Unlike untreated virions, UV-irradiated EMCV failed to induce IL-1β secretion from LPS-primed BMDCs (Figure 2A), indicating that the viral particles or genomic RNAs per se are insufficient to activate the NLRP3 inflammasome. To test whether viral RNA translation is needed to elicit EMCV-induced NLRP3 inflammasome activation, LPS- primed BMDCs were treated with a translation inhibitor cycloheximide (CHX) prior to stimulation with ATP or infection with EMCV. The CHX-pretreated cells produced IL-1β normally in response to extracellular ATP (Figure 2B, left), indicating that NLRP3 inflammasome activation by ATP stimulation does not require de novo translation. This is consistent with a previous report showing that CHX dose not inhibit nigericin-induced NLRP3 activation in primed macrophages [21]. In contrast, pretreatment of cells with CHX significantly inhibited EMCV-induced IL-1β secretion (Figure 2B, right). These data indicate that virus-encoded proteins or viral RNA transcripts, not viral genomic RNAs, activate the NLRP3 inflammasome.

Next, we tested whether viral RNAs were able to activate the NLRP3 inflammasome after infection. To this end, we examined the abilities of viral genomic RNAs and transcripts from EMCV-infected cells to trigger NLRP3 inflammasome activation, by measuring IL-1β secretion from BMMs transfected with these RNAs. Although transfection with RNAs from the virions or virus-infected cells induced robust expression of interferon-β (Figure 2C), it failed to stimulate secretion of IL-1β from BMMs (Figure 2D). It is possible that the amount (50 µg) of transfected viral RNA was not enough to trigger NLRP3 inflammasome activation. However, we ruled out this possibility by measuring the amounts of viral genomic RNA obtained from virions. We collected about 12 µg of viral genomic RNA from 4×10^8^ plaque forming unit (PFU) of virions (Figure 2E). In contrast, we used 1.2×10^6^ PFU of EMCV for the infection at an MOI of 1.5, in which IL-1β secretion was clearly detected (Fig. 1B). Furthermore, since EMCV is a positive single-stranded RNA virus, transfection of cells with viral genomic RNA would produce virions in the supernatant which could stimulate the NLRP3 inflammasome. Indeed, we detected about 600 PFU of EMCV in the supernatant of the cells transfected with 50 µg of viral genomic RNA at 24 h post transfection (Figure 2F). Infection of 8×10^5^ cells with 600 PFU of EMCV corresponds to the MOI below 0.001, which is insufficient to induce IL-1β secretion (Figure 1B). Thus, these results indicate that viral RNA genome and transcripts are insufficient to trigger robust activation of the NLRP3 inflammasome and that the signal 2 is probably derived from virus-encoded proteins.

### EMCV viroporin 2B is sufficient to trigger inflammasome activation

**Figure 3:**
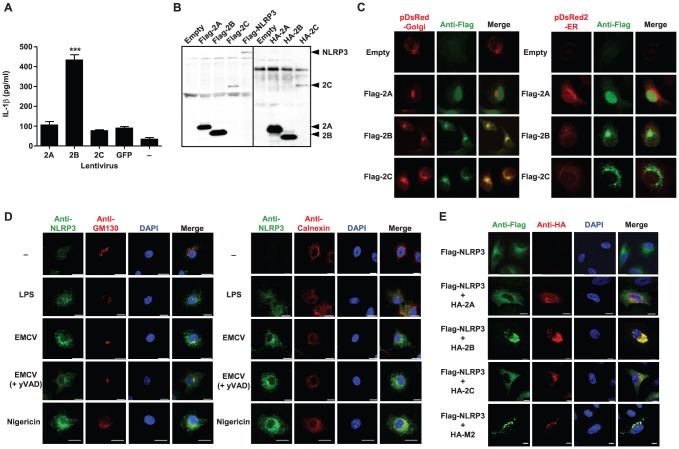
NLRP3 inflammasome activation by EMCV viroporin 2B. (A) LPS-primed BMMs were infected with the lentivirus expressing EMCV 2A, 2B, or 2C, or GFP. Supernatants were collected at 24 h post infection and analyzed for IL-1β by ELISA. (B) HEK293T cells were transfected with the expression plasmid encoding Flag- or HA-tagged EMCV 2A, 2B, or 2C, or NLRP3 or empty vector. Samples were analyzed by immunoblot with rabbit polyclonal antibody against Flag or mouse monoclonal antibody against HA. (C) HeLa cells were transfected with the expression plasmid encoding Flag-tagged EMCV 2A, 2B, or 2C (green) and that encoding either pDsRed-monomer-Golgi or pDsRed2-ER (red), and observed with a confocal microscope at 24 h after transfection. (D) LPS-primed BMMs were stimulated with EMCV or nigercin for 24 h in the presence or absence of yVAD-CHO. Cells were stained with anti-NLRP3 (green) and anti-GM130 (Golgi marker) or anti-calnexin (ER marker) (red) and analyzed by a confocal microscopy. Mock-treated cells were also examined. Nuclei were visualized by staining with DAPI. (E) HeLa cells were transfected with the expression plasmid encoding Flag-tagged NLRP3 (green) and that encoding HA-tagged EMCV 2A, 2B, or 2C, or influenza virus M2 protein (red). Nuclei were visualized by staining with DAPI. Scale bars represent 10 µm. Data are representative of at least three independent experiments, and indicate the mean ± S.D (A). ***, *P*<0.001.

We previously demonstrated that a mutant M2 protein of influenza virus, which has lost its proton selectivity and enabled the transport of other cations (Na^+^ and K^+^), induced enhanced IL-1β secretion compared with the wild-type M2 protein [9]. In addition, picornavirus 2B proteins have been reported to act as viroporins [13]. Thus, we hypothesized that EMCV viroporin 2B protein may trigger inflammasome activation by altering intracellular ionic concentrations. To test this idea, we stimulated BMMs with LPS (signal 1) and transduced them with the lentivirus expressing the EMCV 2A, 2B, or 2C protein. IL-1β was released from LPS-primed BMMs transduced with the 2B-expressing lentivirus, but not from those transduced with other lentiviruses (Figure 3A). These data indicated that the expression of EMCV viroporin 2B is sufficient to activate the NLRP3 inflammasome.

Next, we determined the subcellular localization of EMCV 2B protein responsible for EMCV-induced inflammasome activation by using a confocal microscopy. To this end, we first generated plasmids expressing influenza virus hemagglutinin (HA)- or Flag-tagged proteins and confirmed their expression in HEK293T cells by immunoblot analysis (Figure 3B). When each protein was expressed in HeLa cells, the EMCV 2B and 2C proteins were localized to the Golgi apparatus and other cytoplasmic structures, and the EMCV 2A protein to the nucleus (Figure 3C and Figure S3). We also examined the intracellular localization of NLRP3. In agreement with a previous report [8], stimulation of cells with LPS induced NLRP3 expression in the cytosol (Figure 3D). Upon infection with EMCV, NLRP3 was redistributed to the perinuclear region or cytoplasmic granular structures, which is also observed in cells stimulated with other NLRP3 ligands such as monosodium urate (MSU), alum or nigericin ( Figure 3D ) [22] and considered as a hallmark of NLRP3 activation. Although resting cells or cells expressing the EMCV 2A or 2C protein uniformly expressed NLRP3 throughout the cytoplasm, it was redistributed to the perinuclear region in EMCV 2B- or influenza virus M2-expressing cells (Figure 3E). In addition, the nonstructural 2B proteins from poliovirus and enterovirus 71, members of the genus *Enterovirus* within the family *Picornaviridae*, also induced the redistribution of NLRP3 to the perinuclear region (Figure S4). Notably, the redistributed NLRP3 was largely co-localized with the EMCV 2B, influenza virus M2, poliovirus 2B, and enterovirus 71 2B proteins (Figure 3E and Figure S4). We also demonstrated that NLRP3 was redistributed to the perinuclear region and co-localized with EMCV 2B protein in BMMs transduced with the lentivirus expressing the Flag-tagged EMCV 2B but not 2A or 2C protein (Figure S5). Together, these data provide evidence that the EMCV viroporin 2B alone is sufficient to trigger NLRP3 inflammasome activation and IL-1β secretion from LPS-primed BMMs.

The redistribution of NLRP3 after EMCV infection was not inhibited by yVAD-CHO (Figure 3D). This result is consistent with the activity of yVAD-CHO operating at the step after NLRP3 activation.

### Elevation of the intracellular Ca^2+^ level is required for EMCV-induced IL-1β secretion

**Figure 4:**
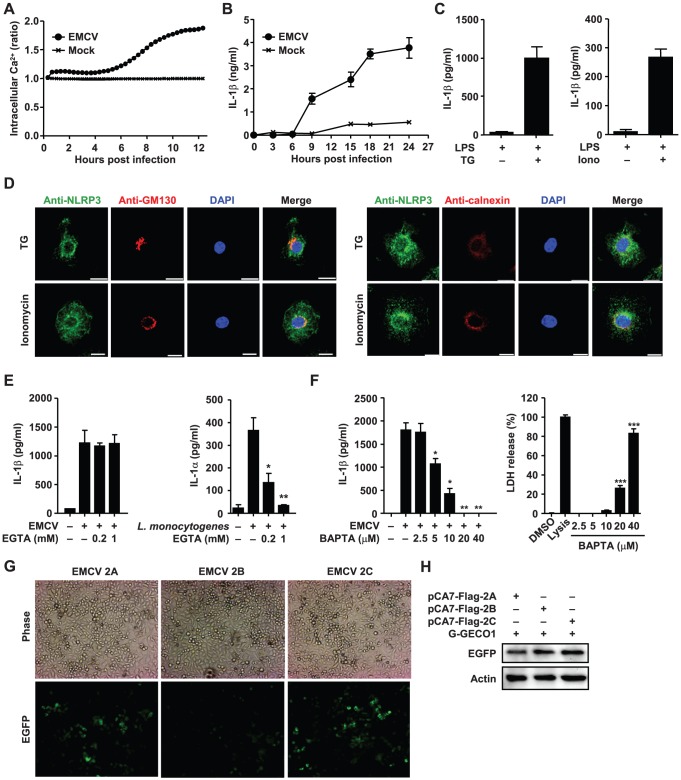
Requirement of increased intracellular Ca^2+^ concentration for NLRP3 inflammasome activation. (A) Intracellular Ca^2+^ concentration in HeLa cells infected with EMCV. The ratio of fluorescent intensities was recorded with an ICCD camera/image analysis system. (B) LPS-primed BMDCs were infected with EMCV. (C, D) LPS-primed BMMs were stimulated with thapsigarsin (TG) or ionomycin. (D) Cells were stained with anti-NLRP3 (green) and anti-GM130 or anti-calnexin (red) and analyzed by a confocal microscopy. Nuclei were visualized by staining with DAPI. (E) LPS-primed BMMs were infected with EMCV or *L. monocytogenes* in the presence or absence of EGTA. (F) LPS-primed BMMs were infected with EMCV in the presence or absence of BAPTA-AM. LDH activity was measured as control for cytotoxicity. (G–H) HeLa cells were transfected with 2.5 µg each of pCA7-Flag-2A, pCA7-Flag-2B, or pCA7-Flag-2C and 0.5 µg of pDsRed2-ER/Golgi-G-GECO1, which encodes the EGFP chimera that is targeted to ER/Golgi and changes its fluorescence levels according to the Ca^2+^ concentrations, and observed under a phase-contrast or fluorescence microscope at 72 h after transfection (G). At 72 h after transfection, samples were analyzed by immunoblot with mouse monoclonal antibody against EGFP or mouse monoclonal antibody against actin (H). Cell-free supernatants were collected at 24 h after stimulation and analyzed for IL-1α or IL-1β by ELISA (B–C, E–F). Data are representative of at least three independent experiments, and indicate the mean ± S.D. (B–C, E–F). *, *P*<0.05; **, *P*<0.01; ***, *P*<0.001.

It has been demonstrated that picornavirus 2B proteins, including EMCV 2B, are mainly localized to the endoplasmic reticulum (ER) and Golgi apparatus, and reduce Ca^2+^ levels in those organelles ([Ca^2+^]_ER_ and [Ca^2+^]_Golgi_) [23], thereby presumably elevating the local concentration of cytoplasmic Ca^2+^ ([Ca^2+^]_cyt_). To examine whether the elevation of [Ca^2+^]_cyt_ by the EMCV 2B protein plays a role in NLRP3 inflammasome activation, we first measured the kinetic changes in the [Ca^2+^]_cyt_ after infection with EMCV by using a calcium-dependent fluorescent probe. Infection with EMCV resulted in a significant increase in the fluorescence intensity of the cells compared with that of mock-infected cells (Figure 4A). The elevation of fluorescence intensity became apparent around 8 hours post infection (Figure 4A), which corresponded to the initiation of IL-1β secretion in the culture supernatant (Figure 4B).

To better understand the role of the elevation of [Ca^2+^]_cyt_ in NLRP3 inflammasome activation, we undertook three different approaches. First, we tested the effects of [Ca^2+^]_cyt_-increasing drugs on IL-1β secretion (Figure 4C and D). Thapsigargin (TG) is a non-competitive inhibitor of the sarco-ER Ca^2+^ ATPase (SERCA) that transports cytoplasmic Ca^2+^ into the ER [24]. Treatment with TG induced IL-1β secretion from LPS-primed BMMs (Figure 4C). Similarly, the Ca^2+^ ionophore ionomycin induced significant release of IL-1β (Figure 4C). When cells were treated with TG or ionomycin, NLRP3 was redistributed to the perinuclear region or cytoplasmic granular structures (Figure 4D), as in EMCV-infected cells (Figure 3D) or EMCV 2B- or influenza virus M2 protein-expressing cells (Figure 3E). Thus, these data indicate that an increase in [Ca^2+^]_cyt_ is important in NLRP3 inflammasome-mediated IL-1β release.

Second, we investigated main sources for Ca^2+^ flux required for EMCV-induced inflammasome activation. Addition of EGTA in the extracellular medium, which blocked ionomycin-induced Ca^2+^ influx (Figure S6) and *Listeria monocytogenes*-induced secretion of IL-1α [25] (Figure 4E, right), had no effect on EMCV- or EMCV 2B-induced inflammasome activation (Figure 4E and Figure S7A). Next, we tested whether the cell-permeable Ca^2+^-chelator BAPTA-AM inhibits EMCV-induced inflammasome activation. The release of lactate dehydrogenase (LDH) from BAPTA-AM treated cells was measured as an index of cytotoxicity. Treatment of BMMs with 5–10 µM of BAPTA-AM (LDH release <3%) significantly blocked IL-1β secretion by EMCV or EMCV 2B protein (Figure 4F and Figure S7B) and the redistribution of the NLRP3 (Figure S8). Furthermore, EMCV 2B protein, but not 2A or 2C protein, specifically reduced [Ca^2+^]_ER/Golgi_ (Figure 4G), as demonstrated using an EGFP chimera that modulates its fluorescence in response to changes in the Ca^2+^ concentration [26]. We confirmed the fluorescence changes of the EGFP chimera after treatment of transfectants with TG (Figure S9) and the comparable expression levels of the EGFP chimera in different transfectants (Figure 4H). These results indicate that Ca^2+^ flux from intracellular storages, but not from extracellular medium, is important in EMCV-induced inflammasome activation, as expected from the function of the 2B protein.

Third, we examined whether Ca^2+^ flux-induced activation of inflammasome requires the action of calpain, a Ca^2+^-dependent cysteine protease. To this end, we examined the effects of carbobenzoxy-valyl-phenylalanial (MDL-28170, calpain inhibitor III) on EMCV-induced inflammasome activation. While 12–100 µM of MDL-28170 significantly blocked *L. monocytogenes*-induced processing of IL-1α [25] (Figure S10B), even 100 µM of the drug had no effect on EMCV- or EMCV 2B-induced inflammasome activation (Figure S7A and Figure S10A). These data indicate that EMCV infection increases [Ca^2+^]_cyt_ via the flux from intracellular storages and thereby activates NLRP3 inflammasome in an calpain-independent manner.

### EMCV activates NLRP3 inflammasome independent of mitochondrial ROS and lysosomal cathepsin B

**Figure 5:**
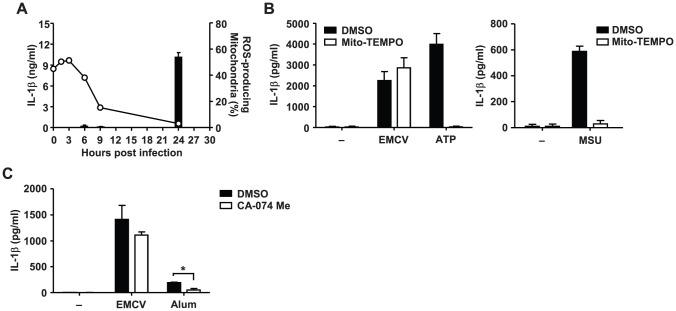
Mitochondrial ROS- and cathepsin B-independent activation of NLRP3 inflammasome by EMCV. (A) LPS-primed BMMs were infected with EMCV. Cell-free supernatants were collected at indicated time points and analyzed for IL-1β by ELISA (left y axis, filled bars). Cells were collected at indicated time points and stained with MitoSOX for 30 min and analyzed by flow cytometry. The proportions of ROS-producing mitochondria are shown (right y axis, open circles). (B–C) LPS-primed BMMs were treated with EMCV, ATP, MSU (B), or Alum (C) in the presence or absence of Mito-TEMPO (500 µM), a scavenger specific for mitochondrial ROS, or cathepsin B inhibitor CA-074 Me (10 µM) (C). Cell-free supernatants were collected at 24 h (EMCV, Alum) or 6 h (ATP) post infection or stimulation, and analyzed for IL-1β by ELISA. Data are representative of at least three independent experiments, and indicate the mean ± S.D. *, *P*<0.05.

We finally tested the role of previously identified factors that can activate the NLRP3 inflammasome [22], [27], [28], [29]. Since mitochondrial ROS was found to be important in NLRP3 inflammasome activation by MSU, alum, and ATP [22], [27], we first measured the kinetic changes in ROS-producing mitochondria after infection with EMCV. The level of ROS-producing mitochondria in LPS-primed BMMs infected with EMCV peaked at 3 h after infection and gradually decreased to the basal level by 24 h (Figure 5A). The kinetics of the change was not correlated with the levels of mature IL-1β in culture supernatants (Figure 5A). Furthermore, treatment with antioxidant Mito-TEMPO, a scavenger specific for mitochondrial ROS [30], [31], had no effect on the secretion of IL-1β in BMMs infected with EMCV (Figure 5B) or transduced with EMCV 2B (Figure S7A), but significantly inhibited IL-1β secretion in response to ATP or MSU (Figure 5B), as reported previously [27].

Cathepsin B, a specific lysosomal cysteine protease, has been implicated in NLRP3-mediated IL-1β production in response to nonviral signals [28], [29] and influenza virus infection [32]. We therefore examined the effect of the cathepsin B-specific inhibitor, Ca-074-Me, on EMCV-induced inflammasome activation. In agreement with a previous report [29], alum-induced release of IL-1β was significantly inhibited by the Ca-074-Me (Figure 5C). In contrast, IL-1β production in response to EMCV or EMCV 2B was not affected in Ca-074-Me-treated BMMs (Figure 5C and Figure S7A). Thus, these data indicate that EMCV activates the NLRP3 inflammasome independent of mitochondrial ROS and cathepsin B.

## Discussion

**Figure 6:**
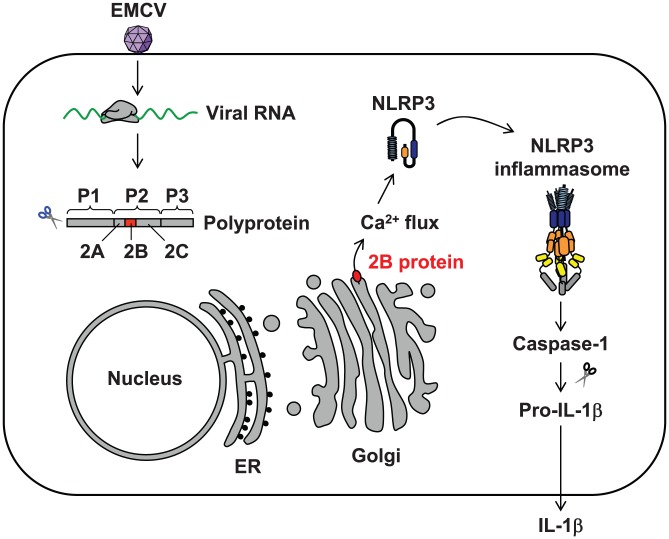
A schematic model of EMCV-induced IL-1β secretion. After infection, the newly synthesized viral polyprotein is cleaved by viral proteases. Within the Golgi apparatus or other cytoplasmic structures, EMCV 2B protein (red) induces Ca^2+^ flux and stimulates the NLRP3 inflammasome pathway. The activated caspase-1 catalyzes proteolytic processing of pro-IL-1β into its mature form, leading to their secretion across the plasma membrane.

In this study, we demonstrated that EMCV, a positive strand RNA virus, activates the NLRP3 inflammasome by increasing [Ca^2+^]_cyt_ through the action of virus-encoded viroporin 2B (Figure 6).

Inflammasome-mediated cytokine release via NLRP3 requires two signals: signal 1 drives pro-IL-1β synthesis while signal 2 induces activation of caspase-1 and cleavage of pro-IL-1β. In the present study, we found that EMCV-induced IL-1β secretion requires LPS priming as signal 1, in agreement with a previous report [20]. This may be partly explained by the fact that EMCV has a strategy to shut off host mRNA translation [33]. LPS signaling may be able to overcome this viral inhibitory activity. In contrast, Poeck et al. have demonstrated that EMCV induces pro-IL-1β synthesis via MDA5 [19]. The reasons for these discrepant results are unclear, but could be different EMCV strains used.

Currently, two distinct inflammasome complexes have been shown to be involved in RNA virus-induced caspase-1 activation (signal 2): the NLRP3 inflammasome and the RIG-I inflammasome. Poeck et al. reported that vesicular stomatitis virus (VSV), a negative strand RNA virus of the family *Rhabdoviridae*, activates RIG-I, which in turn recruits the adaptor ASC and pro-caspase-1 to form the RIG-I inflammasome in an NLRP3-independent manner [19]. In contrast, Rajan et al. showed that like influenza virus [9], VSV activates the NLRP3 inflammasome independently of RIG-I and MDA5 [20]. Measles virus, a negative strand RNA virus of the family *Paramyxoviridae*, which induces type I interferons through its recognition by RIG-I and MDA5 [34], [35], activates the NLRP3 inflammasome but not the RIG-I inflammasome [36]. Our present study also show that IL-1β secretion from EMCV-infected cells is mediated by the NLRP3 inflammasome, consistent with previous reports [19], [20]. Thus, the NLRP3 inflammasome appears to play a central role in caspse-1-dependent IL-1β secretion after RNA virus infections.

Although TLRs and RLHs are known to recognize viral RNAs, it remains unclear whether viral RNAs are also required to activate the NLRP3 inflammasome. In the case of influenza virus, transcriptional induction of genes encoding pro-IL-1β, pro-IL-18, and NLRP3 is activated through TLR7 that recognizes viral RNA, whereas the NLRP3 inflammasome activation is caused by the virally-encoded proton-selective M2 ion channel [9]. Consistent with this observation, Murube et al. showed that treatment of cells with poly(I∶C) failed to elicit inflammasome activation [37]. Similarly, our present study demonstrated that transfection with viral RNAs from EMCV virions or EMCV-infected cells did not stimulate secretion of IL-1β from BMMs, although it induced robust expression of type I interferons. The failure of the viral RNA from EMCV to activate inflammasome activation could be explained by the fact that the adaptor protein ASC specifically interacts with RIG-I but not MDA5 [19]. Furthermore, inactivation of EMCV by ultraviolet irradiation completely abrogated IL-1β secretion, suggesting that viral entry and replication is needed for NLRP3 inflammasome activation. The same findings have been reported for influenza virus [38] and measles virus [36]. Together, these observations suggest that viral genomic RNA, viral transcripts and poly(I∶C) do not act as direct ligands for NLRP3.

Then, how does NLRP3 detect RNA virus infections? The NLRP3 inflammasome can be activated by a wide range of stimuli, such as endogenous danger signals from damaged cells, bacterial components, and environmental irritants, besides viruses [7]. Three models for activation of the NLRP3 inflammasome have been proposed thus far [5], [39], [40]. One model proposes that the binding of extracellular ATP to the purinergic receptor P2X7 on the cell surface plays an important role. Activation of this ATP-gated ion channel triggers K^+^ efflux and the recruitment of pannexin 1 to form a large non-selective pore, which may enable the entry of NLRP3 agonists into the cytosol. In the second model, upon phagocytosis of large crystals and environmental irritants (such as asbestos), lysosomal rupture and cytoplasmic release of lysosomal contents such as cathepsin B may occur in cells, which activates the NLRP3 inflammasome. In the third model, production of ROS from damaged mitochondria may stimulate NLRP3 inflammasome activation [22], [27].

Our data clearly showed that EMCV-induced inflammasome activation is mitochondrial ROS- and cathepsin B-independent. The peak of accumulation of ROS-producing mitochondria in EMCV-infected cells was not correlated with the appearance of mature IL-1β in the supernatant. In addition, EMCV-induced IL-1β was not inhibited by inhibitors of mitochondrial ROS and cathepsin B, which effectively blocked ATP- and Alum-induced IL-1β secretion, respectively. Instead, the elevation of [Ca^2+^]_cyt_ after EMCV infection seemed to be important in NLRP3 inflammasome activation. Picornavirus viroporin 2B proteins have been reported to reduce Ca^2+^ levels in the ER and Golgi apparatus [10], presumably causing Ca^2+^ flux into the cytosol and increasing the local [Ca^2+^]_cyt_. This suggests that the ionic imbalance in the cytoplasm through the action of EMCV 2B may activate the NLRP3 inflammasome. Indeed, we found that EMCV 2B protein specifically reduced [Ca^2+^]_ER/Golgi_, stimulated IL-1β secretion, and induced the redistribution of NLRP3 to the perinuclear region. Notably, the nonstructural 2B proteins from poliovirus and enterovirus 71 also induced the redistribution of NLRP3. Since enterovirus 2B proteins, but not EMCV 2B protein, were found to inhibit protein trafficking, resulting in accumulation of proteins in the Golgi complex [23], the mechanism by which EMCV 2B induces NLRP3 activation could be different from that of poliovirus 2B- and enterovirus 71 2B-induced activation. The notion of the viral nonstructural protein-induced inflammasome activation is indeed supported by our earlier observation that the proton-selective influenza virus M2 protein and its mutant capable of transporting Na^+^ and K^+^ [41], both caused NLRP3 inflammasome activation [9]. Since influenza virus increases [Ca^2+^]_cyt_ [42], [43], [44], it is possible that Ca^2+^ flux into the cytosol also provokes NLRP3 inflammasome activation in influenza virus-infected cells. While further studies are required to better understand how RNA viruses exactly activate the NLRP3 inflammasome, our present study indicates that viroporins and disturbances in the intracellular ionic milieu following viral infections are important in RNA virus-induced NLRP3 inflammasome activation.

Many viruses are known to encode viroporins [13]. For instance, human immune deficiency virus type-1 has an accessory protein Vpu whose transmembrane domain acts as the potassium channel [45], to counteract the host antiviral protein tetherin [46]. The hepatitis C virus p7 protein, a 63-amino acid polypeptide important in assembly and release of infectious virions [47], was found to form hexamers with ion channel activity [48], [49]. Thus, we suspect that in addition to more drastic disruption of membranes by pore-forming toxins [50], [51] and membrane rupture [28], [29], [52], [53], the ionic imbalance via the action of virus-encoded ion channels may be a main target for the NLRP3 inflammasome as a sensor for cellular stress. Knowledge of the exact mechanisms by which NLRP3 detects viruses and thereby affects pathogenesis will provide us with a better understanding of viral diseases to design effective interventions and treatments.

## Materials and Methods

### Ethics statement

Animal experiments were carried out in strict accordance with the recommendations in Guidelines for Proper Conduct of Animal Experiments of Science Council of Japan. The protocol was approved by the Committee on the Ethics of Animal Experiments of the Kyushu University, Japan (Permit Number: A23-087-0). All surgery was performed under sevoflurane anesthesia, and all efforts were made to minimize suffering.

### Mice

C57BL/6 mice used were 8 to 10 weeks of age.

### Cells and viruses

BMMs were prepared as described previously [38]. Briefly, bone marrows from the tibia and femur were obtained by flushing with Dulbecco's modified Eagle's medium (DMEM; Wako Pure Chemical Industry). Bone marrow cells were cultured with DMEM supplemented with 10% heat-inactivated fetal bovine serum (FBS), L-glutamine and 30% L929 cell supernatant containing the macrophage colony-stimulating factor at 37°C for 5 days. For BMDCs, bone marrow from the tibia and femur was obtained as described above, and bone marrow cells were cultured in RPMI 1640 medium containing 10% heat-inactivated FBS, L-glutamine and 5% J558L hybridoma cell culture supernatant containing the granulocyte macrophage colony-stimulating factor (GM-CSF) in 24-well plate for 5 days. The culture medium containing GM-CSF was replaced every other day. RAW264.7 cells, HEK293T cells and HeLa cells were maintained in DMEM supplemented with 10% FBS. PLAT-gp cells (a gift from M. Shimojima and T. Kitamura) containing the retroviral *gag* and *pol* genes [54] were maintained in DMEM supplemented with 10% FBS and blasticidin (10 µg/ml; Invitrogen). 293FT cells (Invitrogen) for lentivirus production were maintained in DMEM supplemented with 10% noninactivated FBS, 6 mM L-glutamine, 0.1 mM non-essential amino acid (NEAA) and 500 µg/ml Geneticin. HT1080 cells were maintained in E-MEM (Eagle's minimal essential medium) supplemented with 10% FBS, 2 mM L-glutamine and 0.1 mM NEAA.

EMCV used for all experiments was grown in L929 cells for 15 h at 37°C. Influenza virus A/PR8 (H1N1) was grown in allantoic cavities of 10 day-old fertile chicken eggs for 2 days at 37°C. Viruses were stored at −80°C, and viral titer was quantified by a standard plaque assay using L929 cells for EMCV and Madin-Darby canine kidney cells for influenza virus. UV inactivation was performed by exposing viruses to 1.0 J of UV light/cm^2^ with a Stratalinker UV closslinker (Stratagene).

### Microorganisms

*L. monocytogenes* EGD (a gift from Y. Yoshikai, Kyushu University) was grown in tryptic soy broth (BD 211825) at 37°C overnight, washed repeatedly, resuspended in PBS containing 10% glycerol, and stored at −80°C in small aliquots. The concentration of bacteria was determined by plating 10-fold serial dilutions of bacterial suspension on tryptic soy agar plates and counting the colonies after cultivation at 37°C overnight.

### Plasmid constructions

The cDNAs encoding EMCV 2A, 2B, and 2C proteins and mouse NLRP3 were obtained by reverse transcription and PCR of total RNA from EMCV-infected L929 cells and LPS-primed RAW264.7 cells, respectively. The cDNAs encoding nonstructural 2B proteins from poliovirus type 1 (Mahoney strain) and enterovirus 71 (SK-EV006/Malaysia/97) were obtained by PCR from their full-length genomic cDNAs (a gift from S. Koike, Tokyo Metropolitan Institute) [55], [56]. These cDNAs were cloned into the eukaryotic expression vectors pCA7 [57] (a derivative of pCAGGS [58]), pCA7-Flag, or pCA7-HA to produce untagged or Flag- or HA-tagged proteins. The cDNA encoding the ER targeting sequence (calreticulin signal sequence) fused to the 5′ end of G-GECO1 were obtained by PCR using specific primers and the pTorPE-G-GECO1 (Addgene), which expresses an EGFP chimera that modulates its fluorescence in response to changes in the Ca^2+^ concentration [26]. The cDNA was cloned into the Nhe I and Bgl II sites of pDsRed2-ER (Clontech) to generate an expression plasmid encoding ER/Golgi-targeted G-GECO1 (pDsRed2-ER/Golgi-G-GECO1).

### Knockdown of genes using shRNA

Target sequences were designed using BLOCK-iT RNAi Designer (Invitrogen) or had been described previously [36]: 5′-GGA TCT TTG CTG CGA TCA ACA-3′ for NLRP3, 5′-GCT CAC AAT GAC TGT GCT TAG-3′ for ASC, 5′-GGA CAA TAA ATG GAT TGT TGG-3′ for caspase-1, and 5′-GGC ACA AGC TGG AGT ACA ACT-3′ for EGFP. To generate shRNA-expressing retroviruses, pRS-shNLRP3, pRS-shASC, pRS-shCaspase-1, and pRS-shEGFP were generated by inserting the DNA fragments containing a mouse RNA polymerase III promoter (from the plasmid pBSsi-mU6 (Takara)) and each target sequence into pRS-U6/puro vector (OriGene). PLAT-gp cells in collagen-coated 10-cm dishes were transfected with 20 µg of each shRNA-expressing pRS vector and 2 µg of pCVSV-G, which encodes the VSV G protein [59], using PEI-Max (Polysciences, Inc). Culture medium was replaced with fresh medium 6 h later, and supernatants containing retroviruses were harvested and filtered through a 0.45-µm filter (Millipore) at 48 h post-transfection. To generate RAW264.7 cells constitutively expressing shRNA targeting NLRP3, ASC, caspase-1, and EGFP mRNA, respectively, RAW264.7 cells were infected with each shRNA-expressing retrovirus in the presence of polybrene (8 µg/ml) at 37°C. Then, cells were washed with phosphate-buffered saline (PBS) and cultured for further 24 h in complete DMEM in the presence of polybrene. Cells were cultured for 2 to 3 weeks in complete DMEM containing puromycin (0.5 µg/ml) to kill non-transduced cells. Levels of expression of targeted genes were analyzed by real-time PCR and Western blot analysis (Figure S2).

### Lentiviral vectors

To generate lentiviruses expressing untagged or Flag-tagged EMCV 2A, 2B, or 2C protein, the full-length cDNA encoding each viral protein was cloned into pLenti6.3/V5-TOPO vector (Invitrogen). 293FT cells in collagen-coated 10-cm dish were transfected with 3 µg of each viral protein-expressing pLenti6.3/V5-TOPO vector together with ViraPower Packaging Mix (Invitrogen) using Lipofectamine 2000 (Invitrogen). Culture medium was replaced with fresh medium 24 h later, and supernatants containing lentiviruses were harvested and filtered through a 0.45-µm filter (Millipore) at 72–96 h post-transfection. Lentivirus encoding an irrelevant protein (GFP) served as a control. The viral titer was quantified using HT1080 cells according to the manufacturer's instructions (Invitrogen). Briefly, aliquots of serial 10-fold dilutions of the stock virus containing polybrene (8 µg/ml) were inoculated into HT1080 cells in six-well plates. Culture medium was replaced with fresh medium 24 h later. Then, cells were cultured in complete medium containing blasticidin (10 µg/ml) to kill non-transduced cells. After 10 to 14 days postinfection, cells were washed with PBS and the number of plaques was counted.

### Infection

BMMs, BMDCs, or RAW264.7 cells were infected with EMCV at an MOI of 1.5 or *L. monocytogenes* at an MOI of 100 for 1 h at 37°C, washed with PBS, and cultured with complete DMEM for 18 to 24 h. Unless otherwise stated, all experiments were performed in LPS-primed BMM, BMDCs, or RAW264.7 cells. To inhibit caspase-1 activation, cells were pretreated with Ac-YVAD-CHO (Bachem) for 30 min. The cells were then infected with EMCV in the presence of Ac-YVAD-CHO for 1 h at 37°C, washed with PBS, and cultured with complete DMEM containing Ac-YVAD-CHO.

### ELISA

Cell-free supernatants were collected at 18–24 h postinfection or transfection with viral RNAs. The supernatants were analyzed for the presence of IL-1α, IL-1β, or IL-6 using an enzyme-linked immunosorbent assay (ELISA) utilizing paired antibodies (eBiosciences) [9].

### LDH release assay

LDH release assays (#G1780, Promega) were performed according to the manufacturer's instructions. LDH release data were used to account for cells death. The data are expressed as percentage of maximum LDH release.

### Flow cytometric analyses

Mitochondria-associated ROS levels were measured by staining cells with MitoSOX (MolecularProbes/Invitrogen) according to manufacturer's instructions. Cells were then washed with PBS, trypsinized, and resuspended in PBS containing 2% FBS. Flow cytometric analysis was performed on a FACSCalibur instrument (BD Bioscience).

### Viral RNA isolation and transfection

Viral RNAs were isolated from virions or L929 cells infected with EMCV at 10 h post infection by using TRIzol reagent (Invitrogen). The RNA extracts were treated with RQ1 DNase (Promega). The concentration of the resultant RNA was determined by NanoDrop technology (Thermo Scientific). BMMs were transfected with total RNA from infected or mock-infected L929 cells, genomic RNA from EMCV, or poly(dA∶dT) (10 µg/ml) using Lipofectamine 2000 (Invitrogen). After 24 h posttransfection, cell-free supernatants were collected and analyzed for the presence of IL-1β using ELISA utilizing paired antibodies (eBiosciences) [9]. At the same time, total RNA was extracted from the cells by using TRIzol reagent (Invitrogen), treated with RQ1 DNase (Promega), and reverse transcribed into cDNAs by using SuperScript III reverse transcriptase (Invitrogen) with an oligo(dT) primer, according to the manufacturer's instructions. The SYBR Premix Ex *Taq* II (Takara) and a LightCycler (Roche Diagnostics, Indianapolis, IN) were used for quantitative PCR with the following primers: mouse IFN-β forward, 5′-GCACTGGGTGGAATGAGACTATTG-3′, and reverse, 5′-TTCTGAGGCATCAACTGACAGGTC-3′; mouse GAPDH forward, 5′-ACCACAGTCCATGCCATCA-3′, and reverse, 5′-TCCACCACCCTGTTGCTGTA-3′.

### Western blot analyses

Subconfluent monolayers of HEK293T or HeLa cells in six-well cluster plates were transfected with pCA7-Flag-2A, pCA7-Flag-2B, pCA7-Flag-2C, pCA7-Flag-NLRP3, pCA7-HA-2A, pCA7-HA-2B, pCA7-HA-2C, or pDsRed2-ER/Golgi-G-GECO1. At 48 to 72 h posttransfection, the cells were washed with PBS and lysed in 1 ml of coimmunoprecipitation buffer [50 mM Tris (pH 7.5), 150 mM NaCl, 1% Triton X-100, 1 mM EDTA, 10% glycerol] containing protease inhibitors (Sigma). The lysates were centrifuged at 20,630×*g* for 5 min at 4°C. Each supernatant was mixed with sodium dodecyl sulfate (SDS) loading buffer [50 mM Tris (pH 6.8), 100 mM DTT, 2% SDS, 0.1% bromophenol blue, 10% glycerol] and boiled for 5 min. These samples were fractionated by SDS-polyacrylamide gel electrophoresis using 12% polyacrylamide gel and electroblotted onto polyvinylidene difluoride (PVDF) membranes (Hybond-P; Amersham Biosciences). The membranes were incubated with anti-Flag (F7425; Sigma), anti-HA (sc-7392; Santa Cruz), anti-GFP (JL-8; Clontech), or anti-actin (sc-8432; Santa Cruz) antibody, followed by incubation with horseradish peroxidase-conjugated anti-rabbit IgG (Invitrogen) or anti-mouse IgG (Invitrogen) for detection of the Flag-tagged or HA-tagged proteins, respectively. The PVDF membranes were treated with Chemi-Lumi One Super (Nacalai Tesque) to elicit chemiluminescent signals, and the signals were detected and visualized using a VersaDoc 3000 imager (Bio-Rad).

### Confocal microscopy

HeLa cells were seeded on coverslips in six-well cluster plates and transfected with 0.5 µg each of pCA7-Flag-2A, pCA7-Flag-2B, pCA7-Flag-2C, pDsRed-monomer-Golgi (Clontech), pDsRed2-ER (Clontech), pCA7-HA-2A, pCA7- HA-2B, pCA7- HA-2C, pCA7- HA-M2, or pCA7-Flag-NLRP3. At 24 h posttransfection, the cells were fixed and permeabilized with PBS containing 2.5% formaldehyde and 0.5% Triton X-100. The cells were then washed with PBS and incubated with anti-Flag (F1804; Sigma) and anti-HA (561; Medical & Biological Laboratories Co.), followed by incubation with Alexa Fluor 488-conjugated donkey anti-mouse IgG (H+L) and Alexa Fluor 594-conjugated donkey anti-rabbit IgG (H+L) (Molecular Probes, Eugene, OR).

To analyze subcellular localization of NLRP3, BMMs were seeded on coverslip in six-well cluster plates and treated with LPS, ionomycin, TG, EMCV, or lentivirus expressing Flag-tagged nonstructural protein from EMCV. At 24 h poststimulation, BMMs were fixed and permeabilized with PBS containing 2.5% formaldehyde and 0.5% Triton X-100. The cells were then washed with PBS and incubated with anti-NLRP3 (Cryo-2, AdipoGen), anti-calnexin (ab22595, Abcam), or anti-GM130 (EP892Y, Abcam), followed by incubation with Alexa Fluor 488-conjugated donkey anti-mouse IgG (H+L) antibodies. Nuclei were stained with DAPI (4.6-diamidino-2-phenylinodole). The stained cells were observed using a confocal microscope (Radiance 2100; Bio-Rad or A1Rsi; Nikon).

### Fluorometric imaging of intracellular Ca^2+^ concentrations

HeLa cells were seeded on 35-mm glass bottom dishes (Matsunami, Osaka, Japan) and infected with EMCV or influenza virus PR8 for 1 h, and washed with PBS and incubated with 1 ml Hanks' balanced saline solution (HBSS) containing 200 mM HEPES and 1 µg/ml Fluo8-AM for 1 h at 37°C. The cells were washed with PBS and incubated in HBSS containing 200 mM HEPES for 30 min at room temperature. Fluorometric cell images were recorded with an ICCD camera/image analysis system, and the intensities were determined. Fluo8-AM fluorescence was measured using excitation at 490 nm and emission at 514 nm.

### Statistics

Statistical significance was tested by one-way ANOVA followed by Tukey's post test using GraphPad PRISM software. P values of less than 0.05 were considered statistically significant.

## Supporting Information

Figure S1 **Caspase-1-dependent IL-1β secretion by EMCV.** LPS-primed BMMs were treated with EMCV, influenza virus PR8 or ATP in the presence or absence of yVAD-CHO. Cell-free supernatants were collected at 24 h after infection and analyzed for IL-1β (A) or IL-6 (B) by ELISA. Data are representative of at least three independent experiments, and indicate the mean ± S.D. **, *P*<0.01; ***, *P*<0.001.
(EPS)

Figure S2 **Expression levels of targeted genes.** (A) Total RNAs were extracted from RAW264.7 cells stably expressing shRNA against NLRP3, ASC, caspase-1, or EGFP mRNAs. mRNA levels of NLRP3, ASC, and caspase-1 were assessed by quantitative RT-PCR. GAPDH was used as an internal control. Data represent the mean ± S.D. of triplicate samples. (B) Samples from RAW264.7 cells stably expressing shRNA against NLRP3, ASC, caspase-1, or EGFP mRNAs were analyzed by immunoblot with either mouse monoclonal antibody against NLRP3, mouse monoclonal antibody against ASC, rabbit polyclonal antibody against caspase-1, or mouse monoclonal antibody against actin. sh control, shRNA targeting an irrelevant mRNA. Indicated below bands are the signal intensities as normalized by that of the corresponding actin. The value in shEGFP-expressing cells was set to 100%. (C) RAW264.7 cells stably expressing shRNA against NLRP3, ASC, caspase-1, or EGFP mRNAs were stimulated with LPS. Cell-free supernatants were collected at 24 h after stimulation and analyzed for IL-6 by ELISA.
(EPS)

Figure S3 **Subcellular localization of nonstructural 2A, 2B, and 2C proteins from EMCV.** HeLa cells were transfected with the expression plasmid encoding Flag-tagged EMCV 2A, 2B, or 2C (green). At 24 h after transfection, cells were stained with anti-GM130 (A) or anti-calnexin (B) and analyzed by a confocal microscopy. Nuclei were visualized by staining with DAPI. Scale bars: 10 µm.
(EPS)

Figure S4 **Redistribution of NLRP3 after transfection with the nonstructural 2B proteins from poliovirus and enterovirus 71.** HeLa cells were transfected with the expression plasmid encoding Flag-tagged NLRP3 (green) and that encoding HA-tagged poliovirus 2B or enterovirus 71 2B protein (red). Nuclei were visualized by staining with DAPI. Data are representative of at least two independent experiments Scale bars: 10 µm.
(EPS)

Figure S5 **The lentivirus expressing EMCV viroporin 2B induces redistribution of NLRP3.** LPS-primed BMMs were infected with the lentivirus expressing Flag-tagged EMCV 2A, 2B, or 2C protein. At 24 h after infection, cells were stained with anti-NLRP3 (green) and anti-FLAG (red) and analyzed by a confocal microscopy. Nuclei were visualized by staining with DAPI. Scale bars: 10 µm.
(EPS)

Figure S6 **EGTA inhibits ionomycin-induced Ca^2+^ influx.** Intracellular Ca^2+^ concentration in HeLa cells stimulated with ionomycin (arrow) in the presence or absence of EGTA in the extracellular medium were recorded with an ICCD camera/image analysis system, and the intensities were determined at indicated time points.
(EPS)

Figure S7 **EMCV viroporin 2B-mediated inflammasome activation depends on caspase-1 and intracellular Ca^2+^ levels.** LPS-primed BMMs were infected with the lentivirus expressing EMCV 2A, 2B, 2C, influenza virus M2, or GFP in the presence or absence of yVAD (50 µM), EGTA (1 mM), MDL-28170 (100 µM), Mito-TEMPO (500 µM), CA-074 Me (10 µM) (A) or BAPTA-AM (10 µM) (B). Supernatants were collected at 24 h post infection and analyzed for IL-1β by ELISA. **, *P*<0.01.
(EPS)

Figure S8 **BAPTA-AM inhibits the redistribution of NLRP3.** LPS-primed BMMs were infected with EMCV for 24 h in the presence or absence of BAPA-AM (10 µM). Cells were stained with anti-NLRP3 (green) and anti-GM130 (red) (A) or anti-calnexin (red) (B) and analyzed by a confocal microscopy. Nuclei were visualized by staining with DAPI. Scale bars: 10 µm.
(EPS)

Figure S9 **Visualization of Ca^2+^ levels with GECOs.** HeLa cells were transfected with the expression plasmid encoding ER/Golgi-G-GECO1 (the EGFP chimera that is targeted to ER/Golgi and changes its fluorescence levels according to the Ca^2+^ concentrations). At 48 h post transfection, cells were stimulated with thapsigarsin (TG) and observed under a phase-contrast or fluorescence microscope.
(EPS)

Figure S10 **EMCV-induced NLRP3 inflammasome activation is calpain-independent.** LPS-primed BMMs were infected with EMCV (A) or *L. monocytogenes* (B) in the presence or absence of MDL-28170. Cell-free supernatants were collected at 24 h after stimulation and analyzed for IL-1β (A) or IL-1α (B) by ELISA. Data are representative of at least three independent experiments, and indicate the mean ± S.D. *, *P*<0.05.
(EPS)
